# Implementation of a three-dimensional (3D) robotic digital microscope (AEOS) in spinal procedures

**DOI:** 10.1038/s41598-022-27082-1

**Published:** 2022-12-29

**Authors:** Stefan Motov, Maximilian Niklas Bonk, Philipp Krauss, Christina Wolfert, Kathrin Steininger, Thomas Picht, Julia Onken, Ehab Shiban

**Affiliations:** 1 Klinik für Neurochirurgie, Kantonsspital St. Gallen, St. Gallen, Switzerland; 2 Klinik für Neurochirurgie, Universitätsklinikum Augsburg, Augsburg, Germany; 3 Klinik für Neurochirurgie, Charité Universitätsmedizin Berlin, Berlin, Germany

**Keywords:** Orthopaedics, Three-dimensional imaging

## Abstract

Three-dimensional exoscopes have been designed to overcome certain insufficiencies of operative microscopes. We aimed to explore the clinical use in various spinal surgeries. We performed surgery on patients with different spine entities in a neurosurgical department according to the current standard operating procedures over a 4-week period of time. The microsurgical part has been performed with Aesculap AEOS 3D microscope. Three neurosurgeons with different degree of surgical expertise completed a questionnaire with 43 items based on intraoperative handling and feasibility after the procedures. We collected and analyzed data from seventeen patients (35% male/65% female) with a median age of 70 years [CI 47–86] and median BMI of 25.8 kg/m^2^ [range 21–33]. We included a variety of spinal pathologies (10 degenerative, 4 tumor and 3 infectious cases) with different level of complexity. Regarding setup conflicts we observed issues with adjustment of the monitor position or while using additional equipment (e.g. fluoroscopy in fusion surgery) (*p* = 0.007/*p* = 0.001). However image resolution and sharpness as well as 3D-depth perception were completely satisfactory for all surgeons in all procedures. The utilization of the exoscopic arm was easy for 76.5% of the surgeons, and all of them declared a significant improvement of the surgical corridor. The 3D-exoscope implementation appears to achieve very satisfactory results in spinal procedures especially with minimally invasive approaches.

## Introduction

Since the introduction of operative microscopes (OMs) in modern microsurgery by Caspar and Yasargil in 1977, there has been a relentless effort to improve visualization, magnification, illumination and ergonomics for the surgeon. Recent studies have criticized limited movement and maneuverability of conventional OMs due to heavy hydraulic counterbalance systems^1,2^. Frequent need for repositioning and fatigue resulting from enforced fixation of the surgeon’s eyes to the OM eyepieces, as well as the limited visualization only for the operating and assisting surgeons, have been pointed out as major shortcomings^2^. In contrast, modern exoscopes have been designed to overcome those disadvantages and, in previous studies, were demonstrated to have comparable three-dimensional visualization with superior ergonomics and enhanced field of view, allowing for even better positioning of surgical instruments^2–4^. In a current review of the usage of exoscopes in neurosurgery, Montemurro et al. describe the current exoscopic systems on the market—VITOM^®^ (Karl Storz, Tuttlingen, Germany), ORBEYETM (Olympus, Tokyo, Japan), Modus VTM (Synaptive Medical, Toronto, ON, Canada), Kinevo 900 (Carl Zeiss Meditec AG, Jena, Germany), BrainPath^®^ (Nico Corporation, Indianapolis, IN, US) and Aeos^®^ (Aesculap, Tüttlingen, Germany)^5^. The authors postulate that those systems provide a more convenient, high-resolution visualization without compromising the surgical corridor. Some modern features include the lock-on-target and waypoints together with the footswitch which enables to place the camera and to return to saved targets of interest, even hands-free. These functions together with the mostly horizontal visual axis of the surgeon and assistant fixed on the monitors during surgery allow for more ergonomic posture and integration of the whole OR staff during the procedure^6,7^. Also the possibility of 3D vision for all OR staff members enables the utilization for education purposes^8,9^. The implementation of the novel Aeos three-dimensional (3D) robotic digital microscope (Aesculap, Tüttlingen, Germany) has been previously displayed in the clinical and experimental setting^3,10,11^. We aimed to investigate the overall use and application in spinal procedures.

## Methods

### Ethics approval

The local ethics committee of Ludwig-Maximilians university approved the study protocol in accordance with the Declaration of Helsinki (local ethics nr. 22-0101).

### Study design

We performed a prospective observational analysis of patient- and procedure-specific clinical data and questionnaires consisting of 43 items using a 5- to 7-point Likert scale (Table 1), which have been previously described^3,12^. Three neurosurgeons with different levels of surgical experience and skills (A = spine professional; B = consultant; and C = resident with an average of 9 years [CI 6–13] of professional experience) were formerly introduced to the Aeos robotic digital microscope by the manufacturer prior to first use and were advised to perform all spine procedures with the system of interest between October 1–31, 2021. Following each procedure the surgeons completed questionnaires, which contained information on the intraoperative satisfaction in terms of image quality, ergonomics, usability and fatigue. All data was collected, encrypted, processed and analyzed according to the study protocol.

**Table 1:** Surgeons’ questionnaires classified in 4 categories.

Task load	Mental demands: Fatigue (very low 1–7 very high)
	Physical Demands: Fatigue (very low 1–7 very high)
	Satisfaction with performance (Not very satisfied 0–7 very satisfied)
	Fatigue (no fatigue at all 0–7 a lot of fatigue)
	Level of Frustration (very low 0–7 very high)
	Level of Distraction (very low 0–7 very high)
	How hurried or rushed was the pace of the procedure? (very low 0–7 very high)
	How complex was the procedure? (not very complex 0–7 very complex)
	How anxious did you feel while performing the procedure? (not very anxious 0–7 very anxious)
General form	Surgical Time (minutes)
	Microsurgery Time (minutes)
	Anaesthesia Time (minutes)
	Use of intraoperative Imaging (0 = no, 1 = yes)
	Photo Documentation (0 = no, 1 = yes)
	Video Documentation (0 = no, 1 = yes)
	Exoscope Recording (0 = no, 1 = yes)
	Case complexity (1 = Low, 2 = Relatively low, 3 = Medium, 4 = Increased, 5 = High, 6 = Very High)
	Patient positioning (1 = supine, 2 = prone, 3 = Lateral)
Utilization and image quality	Distance of 3D Monitor (cm)
	Angle of 3D Monitor (degrees)
	Monitor used (32 “/ 55 “)
	Setup conflicts (No = 0, Yes = 1)
	Adjustments of monitor position (No = 0, Yes = 1)
	Conflicts with additional equipment (No = 0, Yes = 1)
	Integration of surgical assistant (No = 0, Yes = 1)
	Room light condition (Bright = 1, Dim = 2, Dark = 3)
	3D depth perception (Not satisfactory = 0, Satisfactory = 1)
	Image resolution (Not satisfactory = 0, Satisfactory = 1)
	Image sharpness (Not satisfactory = 0, Satisfactory = 1)
	Depth of field (Not satisfactory = 0, Satisfactory = 1)
	Image contrast (Not satisfactory = 0, Satisfactory = 1)
	Color fastness (Not satisfactory = 0, Satisfactory = 1)
	Image luminance (Not satisfactory = 0, Satisfactory = 1)
	Image magnification (Not satisfactory = 0, Satisfactory = 1)
	Visual artefacts (No = 0, Yes = 1)
	Eyestrain (No = 0, Yes = 1)
	Use of 3D glasses
Self assessment	Control of exoscopic arm easy (1 = strongly disagree, 2 = disagree, 3 = neutral, 4 = agree, 5 = strongly agree)
	Surgical working zone unblocked (1 = strongly disagree, 2 = disagree, 3 = neutral, 4 = agree, 5 = strongly agree)
	Surgical ergonomics agreeable during utilization (1 = strongly disagree, 2 = disagree, 3 = neutral, 4 = agree, 5 = strongly agree)
	Surgical hand–eye coordination was affected (1 = strongly disagree, 2 = disagree, 3 = neutral, 4 = agree, 5 = strongly agree)
	Image quality: 3D Perception was satisfactory (1 = strongly disagree, 2 = disagree, 3 = neutral, 4 = agree, 5 = strongly agree)
	Image quality: eyestrain was affecting surgical performance (1 = strongly disagree, 2 = disagree, 3 = neutral, 4 = agree, 5 = strongly agree)

### Patient selection

All patients older than 18 years old who were undergoing a spinal procedure with the Aeos exoscope regardless of spine pathology were enrolled. Procedure-related information, such as patient positioning, operation/anesthesia time (OT/AT), blood loss (BL) and complications, were obtained.

### Statistics

Statistical analysis was performed using the software SPSS Statistics™ (version 28, IBM Corp, Armonk, New York, USA). Descriptive statistics were performed for the clinical parameters and the questionnaire items data. Normal distribution was assumed according to the central limit theorem. In categorical variables, a Levene test for independent samples and a Chi-squared-test was used to compare two samples. Data in text and graphs are shown as mean standard deviation (SD). A *p* value ≤ 0.05 was considered significant.

### Ethical approval and consent to participate

The study was conducted according to the guidelines of the Declaration of Helsinki and approved by the Ethics Committee of the Ludwig Maximilian University (LMU) (project Nr. 22-0101). We retrospectively analyzed prospectively collected data from a patients cohort treated at our clinics with the AEOS exoscope. Based on the retrospective character, informed consent was waived by the local ethics committee of LMU (Prof. Dr. R. M. Huber).

## Results

### Study population and surgical procedures

**Figure 1:**
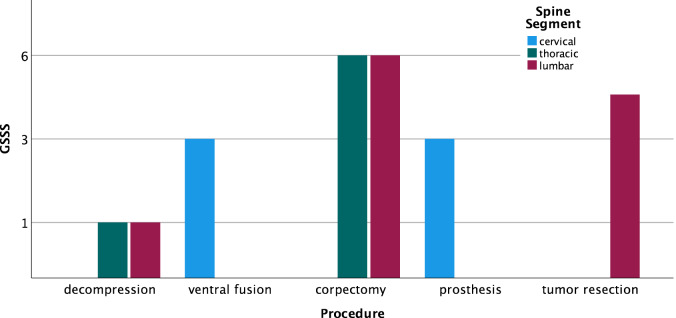
Case complexity based on spine segment and GSSS.

**Figure 2:**
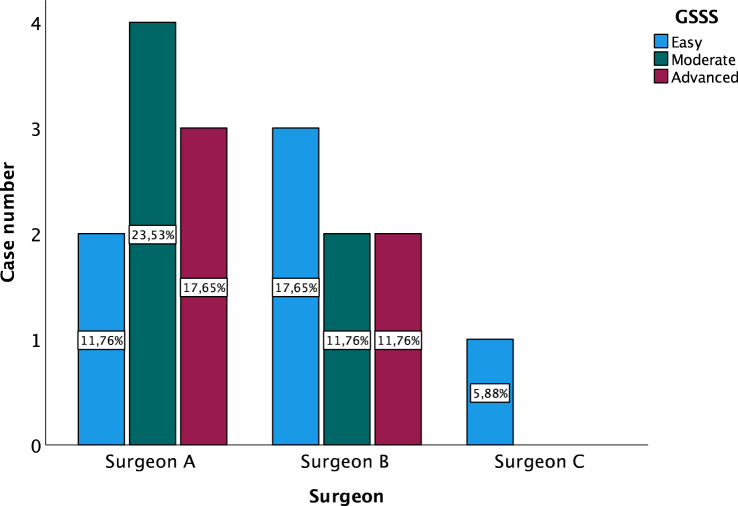
Case complexity distribution between surgeons based on GSSS.

Seventeen patients (35% male/65% female) with a mean age of 68.8 years [CI 47–86] and mean BMI of 25.8 kg/m^2^ [CI 21–33] were included in the final analysis. The indications varied for 10 degenerative (59%), 4 tumor (23%) and 3 infectious (18%) cases with different levels of complexity according to the German Spine Society Score (GSSS) (Table 2; Fig. 1). We performed 6 lumbar decompressions with or without discectomy, 3 anterior cervical discectomies with fusion, 4 thoracolumbar corpectomies via minithoracotomy or retroperitoneal approach, 3 cervical total disc replacements and 1 tumor resection via anterior lumbar approach. Based on the GSSS, there was a balanced case complexity distribution with 35% basic (Score 1), 35% moderate (Score 3) and 30% advanced (Score 6) procedures. Although Surgeon A performed more cases (52%) and higher complexity cases (41%), there was no significant difference considering the overall case complexity distribution (*p* = 0.29) (Fig. 2).

**Table 2:** GSSS description.

Level	Basic	Moderate	Advanced
Spine procedure	1–2 level/-s of lumbar decompression Lumbar discectomy	Anterior cervical discectomy and fusion (ACDF) /total disc replacement (TDR) Posterior instrumentation or spondylodesis	Extreme lateral interbody fusion (XLIF) Complex spondylodesis or spine reconstruction > 3 levels Tumor resection
Score	1	3	6

### Outcome and adverse events

**Figure 3:**
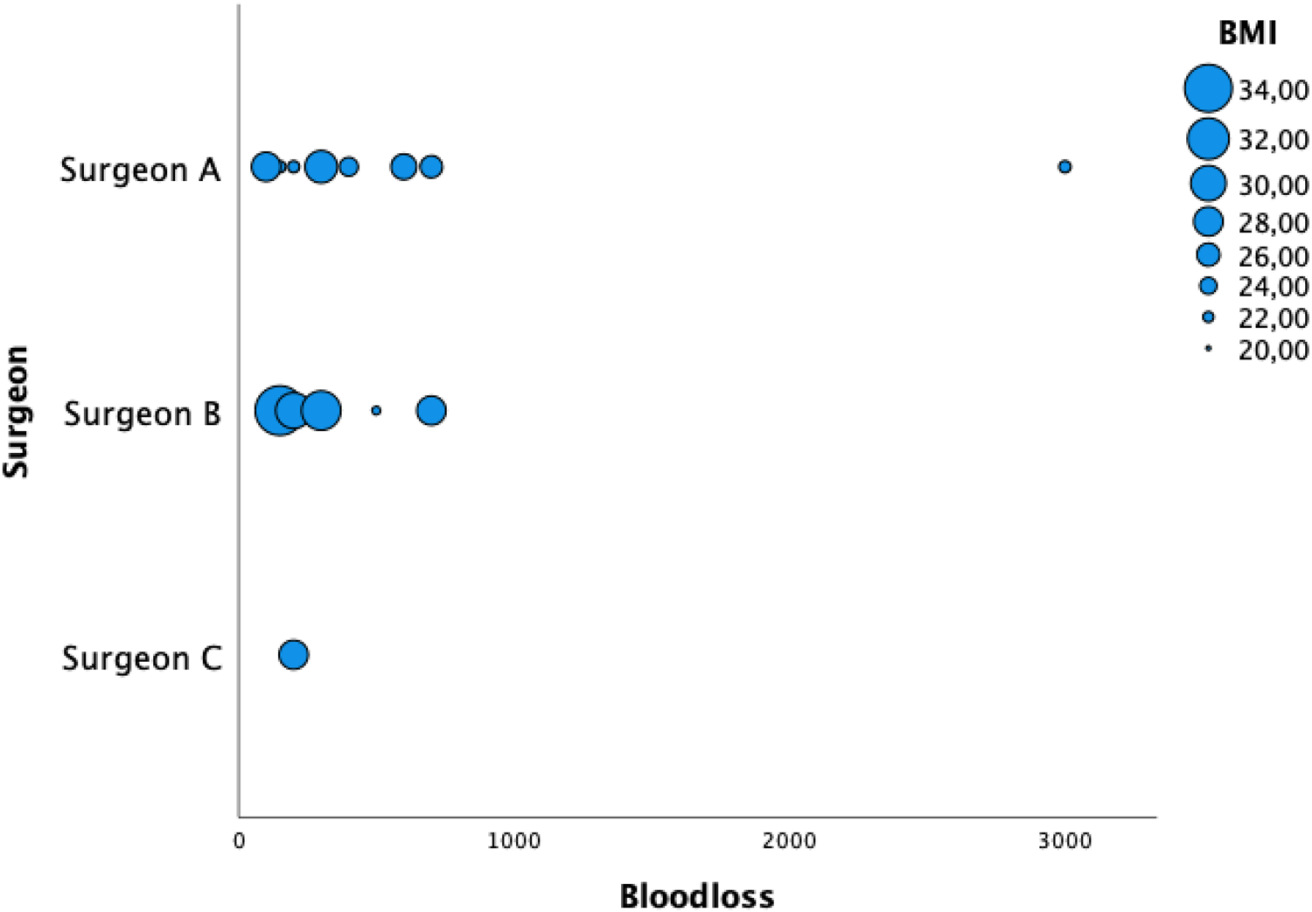
Blood loss distribution based on BMI and surgeon.

**Figure 4:**
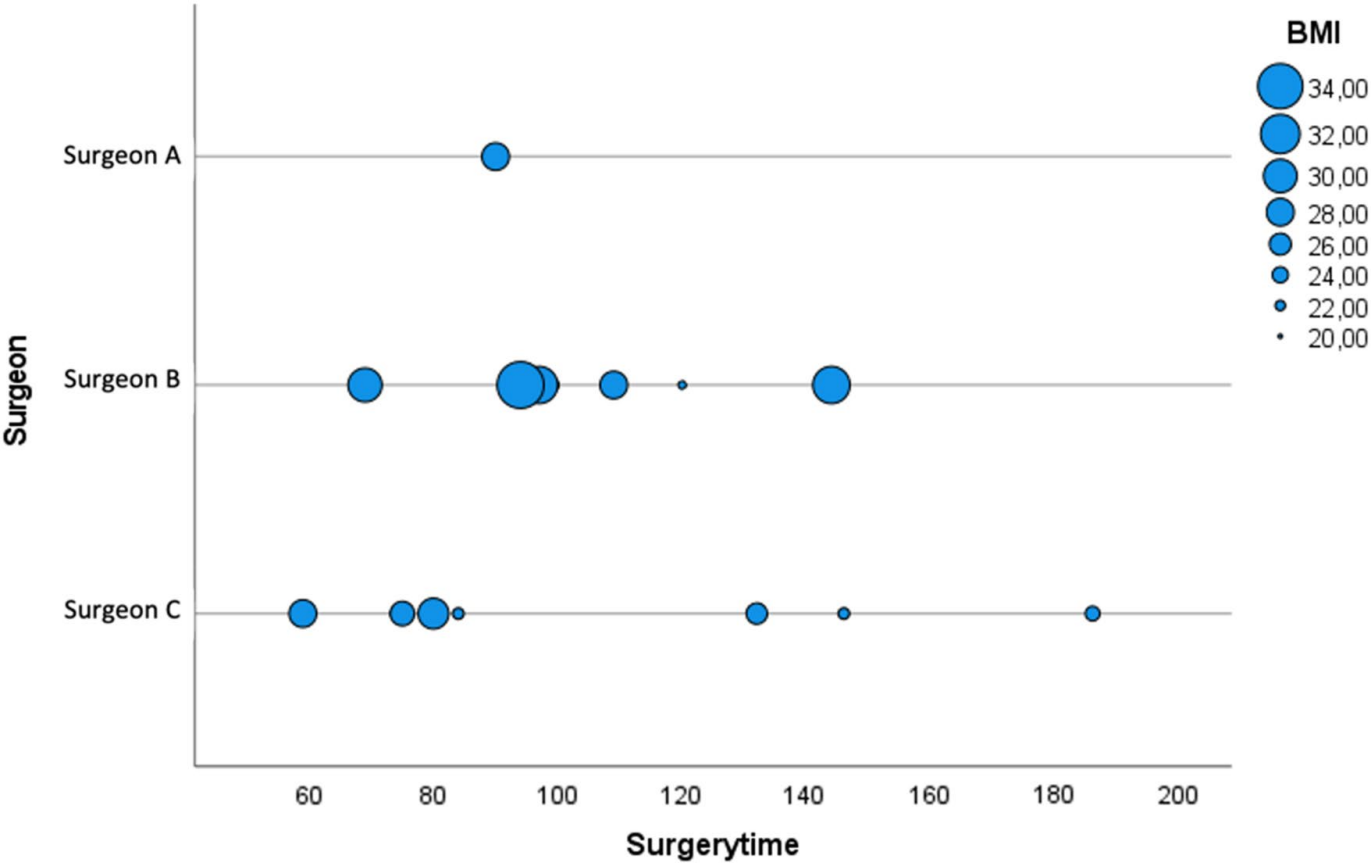
Surgery times (minutes) based on BMI and surgeon.

Mean OT was 102 min [CI 59–186], and mean AT was 163 min [CI 105–319]. Mean BL was 491 ml [CI 100–3000 ml], with 47% of all cases not exceeding a BL of 200 ml. Higher BL (mean 1200 ml) was especially observed in tumor surgery (24%) but without statistical significance (*p* = 0.22) due to inhomogeneous distribution based on diagnosis. Only 1 patient (6%) experienced a complication due to a significant BL (3000 ml), which occurred during a L2 corpectomy indicated for vertebral metastasis of renal cell carcinoma origin, although a preoperative embolization had been performed. The vertebrectomy has been aborted after vertebral body resection due to critical hypotension, and the patient has been packed and transferred to ICU. An uneventful second look surgery with permanent implant insertion was successfully performed 7 days later. In terms of BMI, there was no statistical correlation to a higher blood loss (*p* = 0.43) or prolonged surgical times (*p* = 0.59) (Figs. 3, 4). No neurological deterioration and no system specific complications were observed during the procedures.

### Questionnaire evaluation

#### Setup management

**Figure 5:**
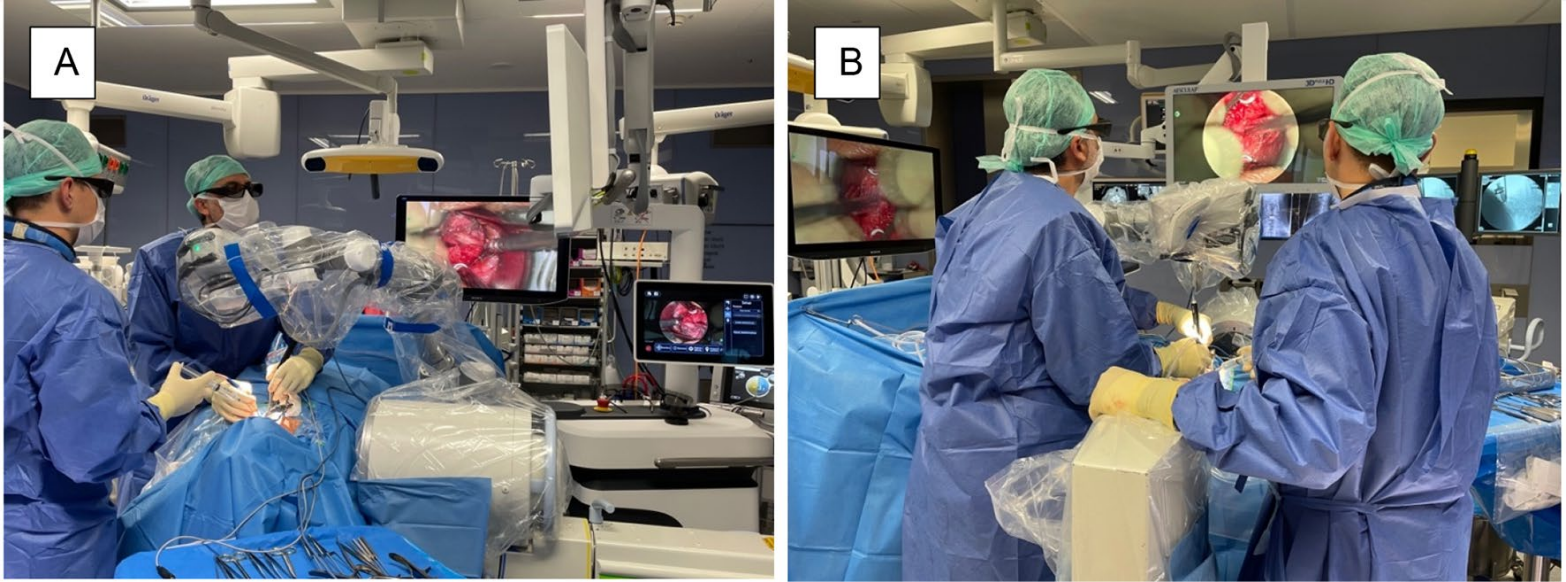
OR setup for ACDF: Surgical corridor remains unblocked with a good visualization for both surgeon and assistant. (**A**) Assistant‘s and OR nurse‘s 55 inch monitor is directly opposite of them; (**B**) 32 inch monitor is in front of the surgeon, fluoroscope is placed in lateral position

**Figure 6:**
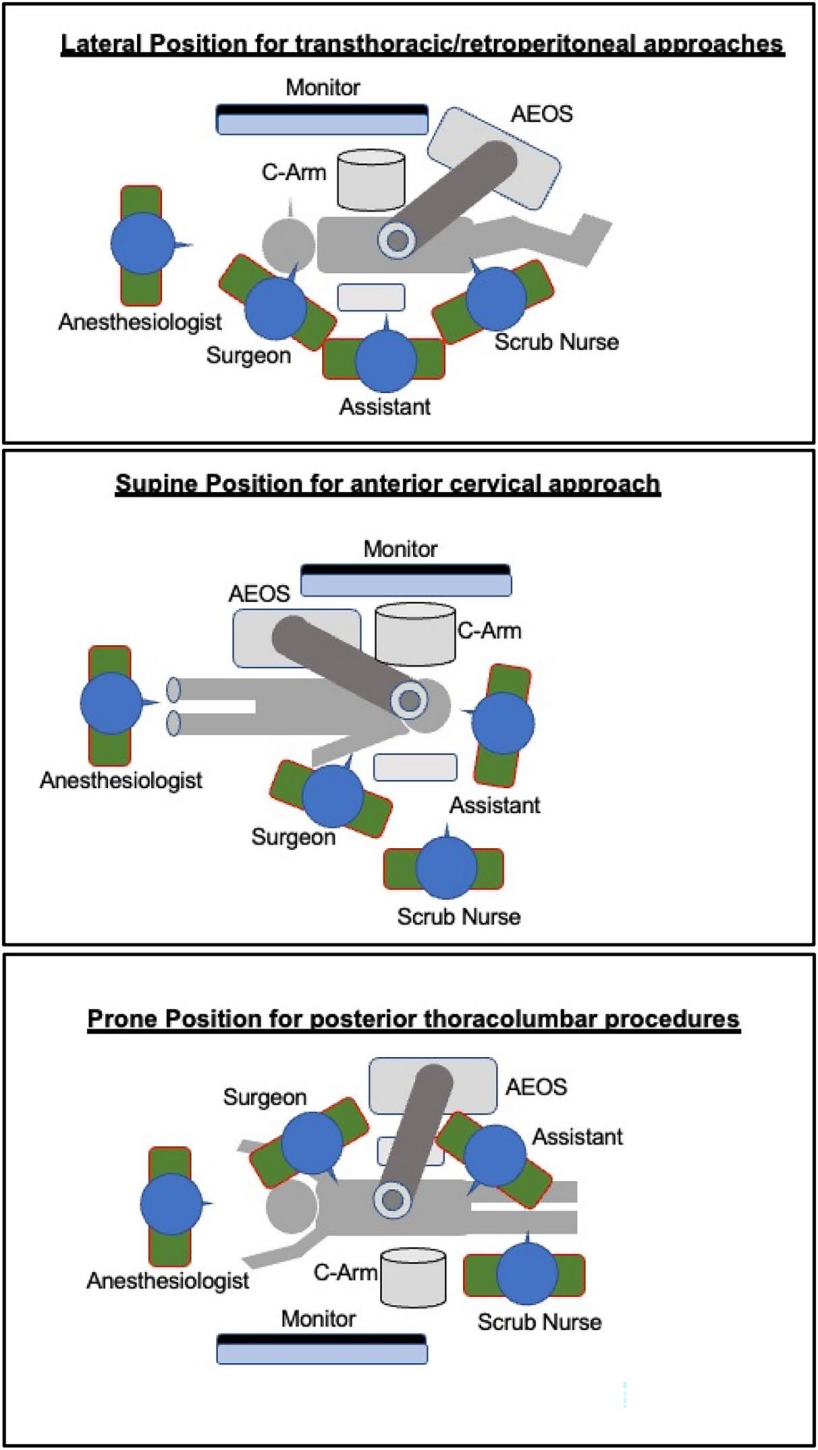
OR setup for cervical and thoracolumbar procedures based on patient and equipment positioning.

**Figure 7:**
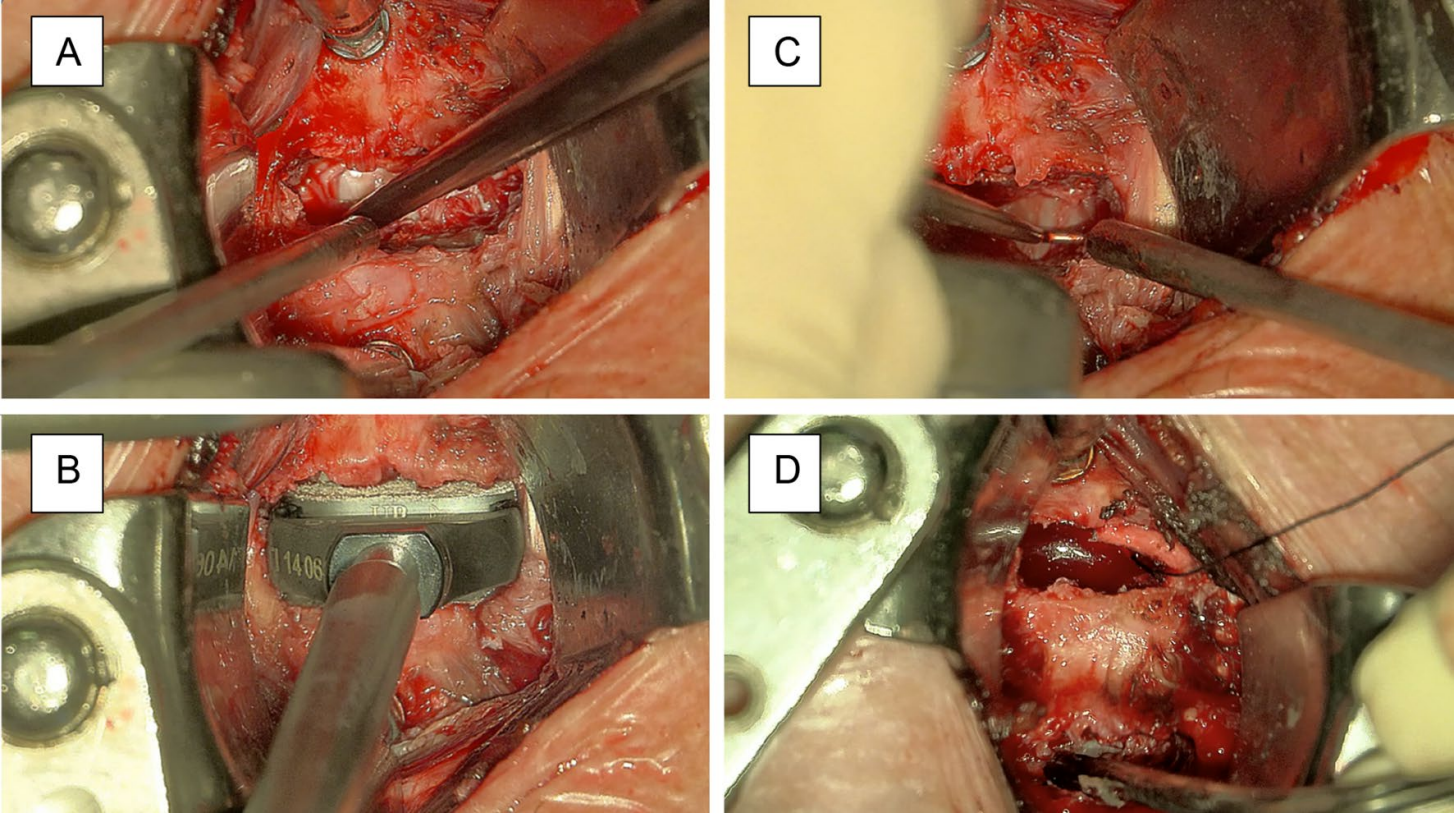
Exemplary case 1: anterior cervical discectomy and disc replacement or fusion. (**A**) Anterior discectomy and removal of the anterior longitudinal ligament; (**B**) Cervical disc replacement with prosthesis; (**C**) Cervical foraminotomy; (**D**) Saving two different targets of focus enables semi-automatic adjustment between the two levels of interest

**Figure 8:**
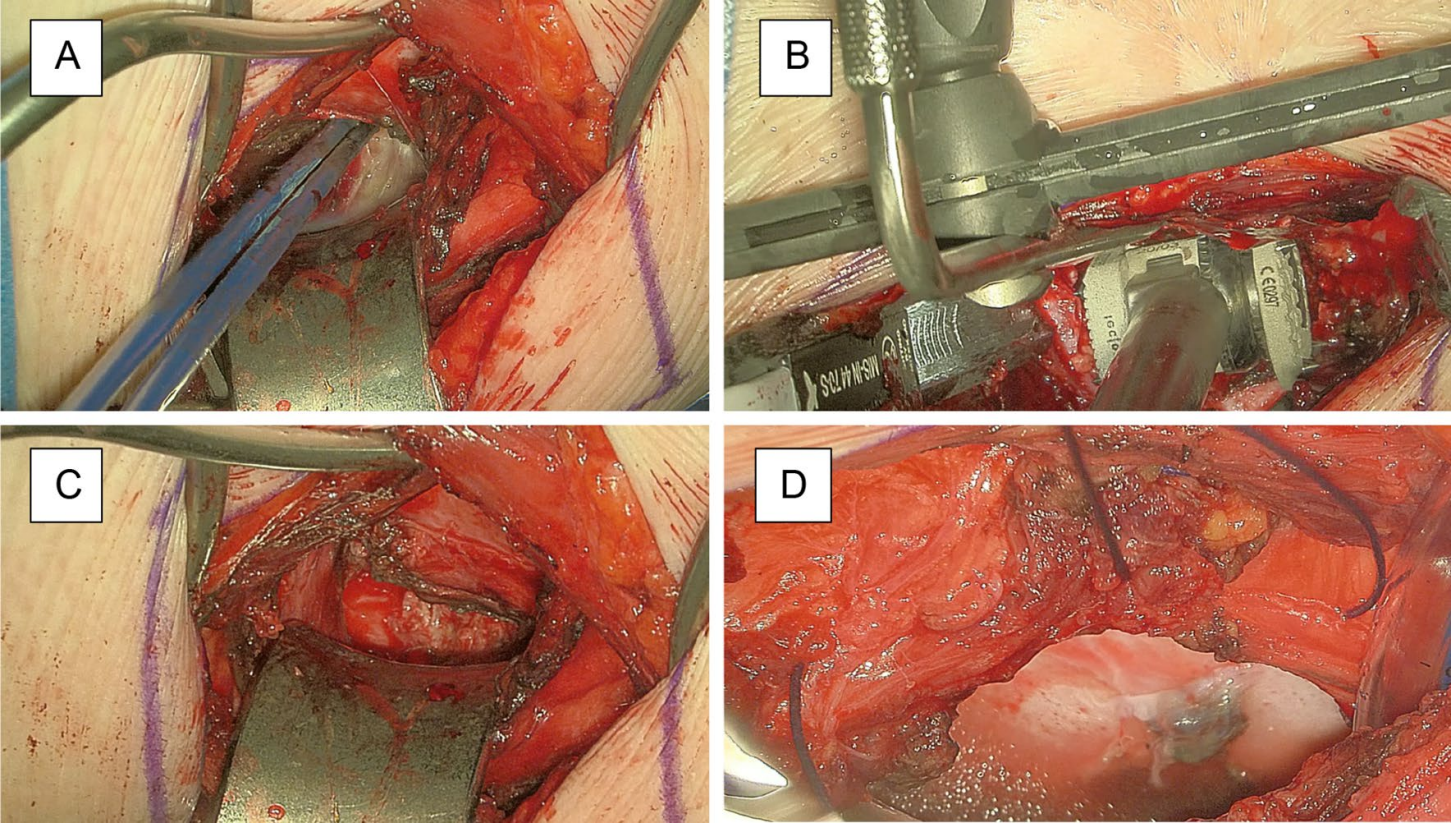
Exemplary case 2: thoracic corpectomy and vertebral body replacement with a carbon fiber-reinforced PEEK implant for metastatic disease. (**A**) Transpleural minithoracotomy approach for vertebral body resection; (**B**) Vizualization of vertebral body replacement; (**C**) Providing different angles of view in the depth enables adequate preparation and hemostasis; (**D**) Watertight closure of pleural cavity after chest tube placement

**Figure 9:**
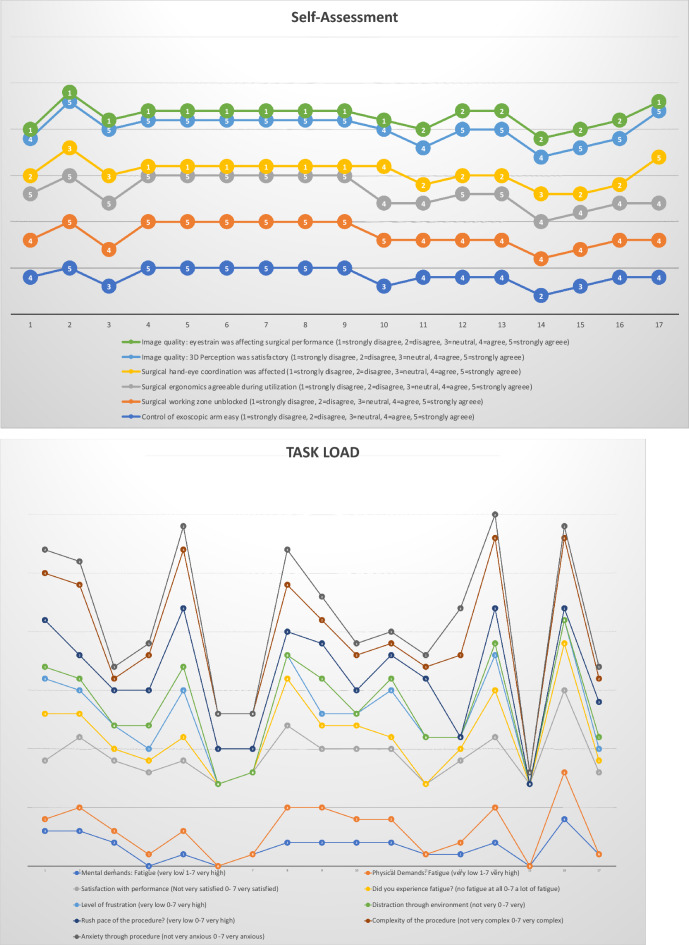

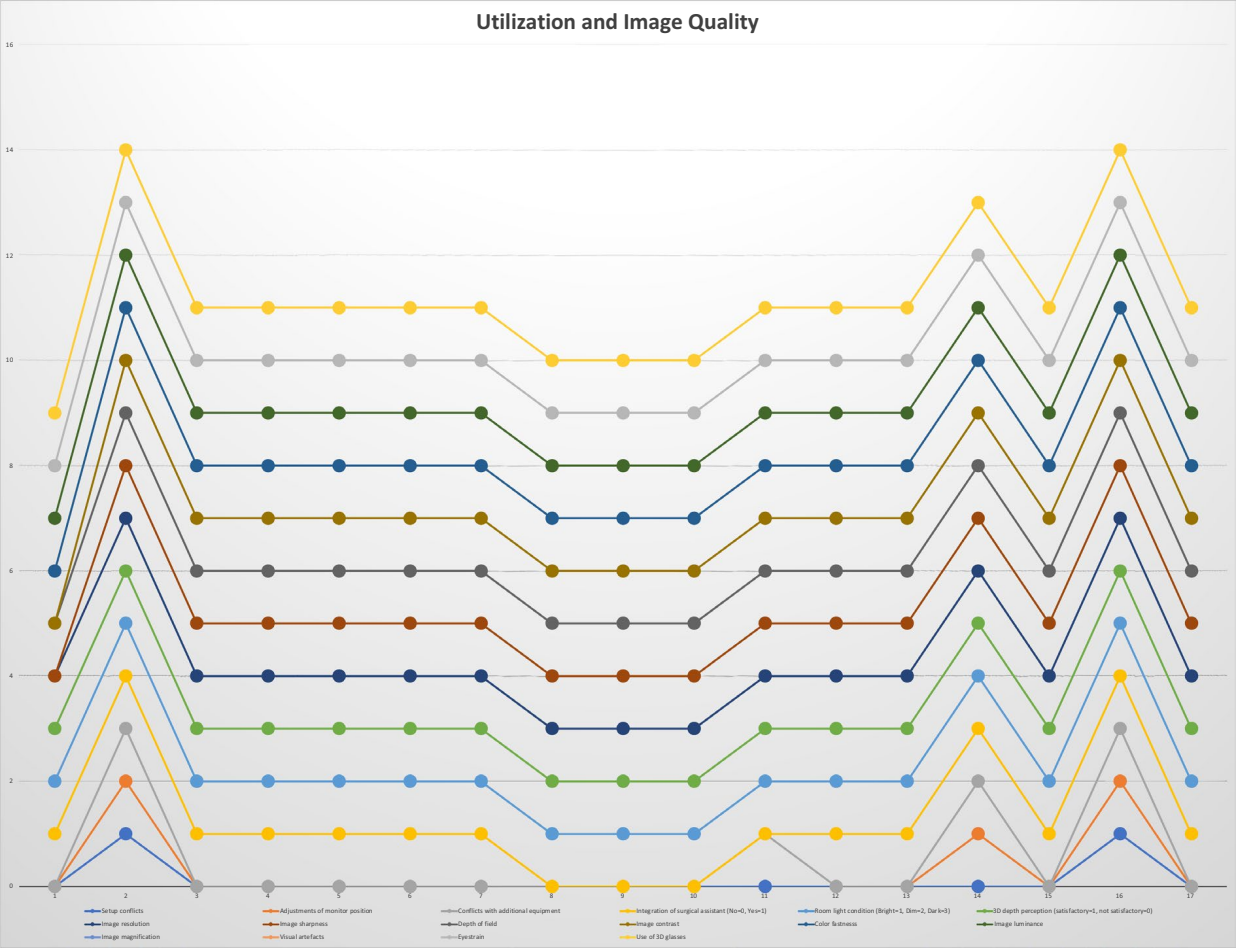
Results of surgeons’ questionnaires classified in the categories self-assessment, task load and utilization and image quality.

In all procedures, surgeons either strongly (47%) or at least agreed (53%) that the surgical zone remained unblocked during surgeries (Figs. 5, 9). In 76% of all cases, the endoscopic arm was easy to control, and only 1 case appeared to be particularly challenging. Setup conflicts occurred in 12% of procedures, which we observed during surgeries in the lateral position, as both the fluoroscope and the exoscope were installed from the opposite site of the surgeon and assistant and had to be adjusted in order to obtain satisfying imaging. We further characterized the recommended OR setup for cervical and thoracolumbar procedures based on the patient and equipment positioning (Fig. 6). Solely the positioning for transthoracic and retroperitoneal approaches used to be more complicated due to the need of frequent fluoscopic control of the spine level and implants. This was particularly difficult as the exoscope and C-Arm gantry were installed on the same side anterior to the patient’s body. Therefore we used the exoscope for the opening, marked the exact position and removed it while installing the retractor and performing the discectomy and contralateral release. Afterwards we placed it once again in the same position for the vertebrectomy and removed it when the cage was in situ. The permanent cage expansion was performed under fluoscopic control and direct visualization. Even if it appeared to be challenging with a mean time prolongation of 34 min [CI 25; 43] in the first 2 cases, we did not abort the use of the exoscope and performed all surgeries without additional OM application. All surgeons agreed that surgical ergonomics during utilization was maintained, but surgical hand–eye coordination was affected in only 11% of all cases. The 3D image quality perception was defined as the perception of the particular surgeon for surgical depth and 3D image quality during the application of the 3D goggles and the exoscopic system. All participants felt that 3D image quality perception was satisfactory in all cases (Fig. 9). Eyestrain was evaluated based on the subjective perception of the surgeon during the use of the 3D goggles and did not occur in all cases. Surgeons stated that switching between different targets of view through the robotic arm’s automatic movement and focus adjustment was highly beneficial throughout complex multilevel procedures (Figs. 7 and 8 Exemplary cases, Fig. 9).

#### Satisfaction

All surgeons rated the surgical satisfaction in 88% of all cases as high (41%) or very high (47%). In the remaining 12%, it was slightly above expectations. In 17.6% of all cases, surgeon’s frustration was above expectations, which was independent from the case complexity based on the GSSS scores (*p* = 0.34) and the surgeon’s experience (*p* = 0.16). We performed an ANOVA test to investigate the influence of further parameters e.g. surgical times, blood loss, spine level, age and BMI and could not identify a significant dependency for the level of frustration based on these values (Table 3). Mental and physical demands were below average in 82% and 76% of all cases, respectively. In only one case, surgeons indicated an above average level of demand, which was the previously mentioned vertebrectomy case for metastatic disease.

**Table 3:** Mean/Standard deviation and ANOVA test for frustration based on surgery times, bloodloss, spine level, age and BMI.

Frustration		Surgery time	Blood loss	Level	Age	BMI
0	Mean	71.00	200.00	4.33	80.67	25.67
	N	3	3	3	3	3
	SD	10.82	100.00	1.15	2.08	3.21
1	Mean	105.80	340.00	6.20	66.20	26.20
	N	5	5	5	5	5
	SD	45.40	238.22	2.17	16.93	4.32
2	Mean	109.25	412.50	7.50	69.75	26.25
	N	4	4	4	4	4
	SD	16.05	332.60	2.38	16.84	2.99
3	Mean	132.00	400.00	5.50	75.50	25.50
	N	2	2	2	2	2
	SD	16.97	141.42	4.95	7.78	6.36
4	Mean	96.67	1200.00	3.67	55.67	25.00
	N	3	3	3	3	3
	SD	42.83	1562.05	1.53	8.02	3.61
Total	Mean	101.94	491.18	5.65	68.82	25.82
	N	17	17	17	17	17
	SD	33.78	679.89	2.49	14.28	3.47

		Square sum	df	Quadratic mean	F	Significance
**ANOVA test**						
Surgery time * frustration	Between the groups	5050.73	4	1262.68	1.15	0.38
	In the groups	13,208.22	12	1100.69		
	Total	18,258.94	16			
Bloodloss * frustration	Between the groups	1,917,301.47	4	479,325.37	1.05	0.42
	In the groups	5,478,875.00	12	456,572.92		
	Total	7,396,176.47	16			
Level * frustration	Between the groups	32.25	4	8.06	1.43	0.28
	In the groups	67.63	12	5.64		
	Total	99.88	16			
Age * frustration	Between the groups	1067.09	4	266.77	1.46	0.28
	In the groups	2195.38	12	182.95		
	Total	3262.47	16			
BMI * frustration	Between the groups	3.75	4	0.94	.06	0.99
	In the groups	188.72	12	15.73		
	Total	192.47	16			

#### Limitations

In this study, we collected limited data in a single institution based on a short time application of a novel 3D exoscope. A higher number of treated patients and a comparative cohort treated with OM would have enabled a randomization and probably more significant results. Long-term follow-up data turns out to be missing in this study particularly considering implant subsidence and tumor or infection recurrence. However, we demonstrated highly satisfying results in several minimally invasive keyhole spine approaches independent from the surgeon’s experience when using the 3D exoscope.

## Discussion

All procedures in this study were performed entirely with the AEOS exoscope, and there was no case in which surgeons switched to the OM due to case complexity. Although surgeons in our institution were only briefly instructed in the utilization of the new exoscope system, they quickly felt comfortable and confident using it even in complex procedures. The Aeos exoscope generates unobstructed access, allowing abundant space to insert and use even longer surgical instruments, which appears to be highly beneficial especially in minimally invasive lateral spine surgery. Giorgi et al. demonstrated in a series of patients with thoracolumbar fractures with spinal cord compression a crucial improvement of the user-friendliness and visualization in lateral thoracolumbar procedures through the possibility to simply shift between microscopic and macroscopic vision without moving the exoscope and without blocking the deep surgical field^13^. Although we experienced some surgical time prolongation in the first two lateral cases, we quickly established an OR setup for all spine procedures based on the patient positioning, which diminished the setup conflicts and allowed a better positioning of the C-Arm.The exoscopic systems already demonstrated satisfying magnification and stereoscopic vision even in more challenging and extensive deformity correction procedures^14^. Still the major advantages, which we also observed in our study, are the improvement of 3D depth perception and magnification in minimally invasive procedures like the classical anterior cervical approach and the lateral thoracolumbar minithoracotomy or the retroperitoneal approach. Yao et al. even demonstrated shorter surgical times and fewer complications in anterior cervical discectomies with fusion due to more adequate ipsi- and contralateral decompression with reduced time of focusing and higher maneuverability and ergonomics^15^. Also the exoscope facilitates a long working distance, which allows surgeons to perform bimanually with standard instruments without conflict with the camera and the use of additional monitor makes it possible for all participants to be better integrated in the procedure^15^. Surgeons in our study confirmed that the robotic arm allowed better control with superior ergonomics and less fatigue compared to the OM. The ergonomics and feasibility were rated high, which correlates with the experience in previous studies^3,4,12^. Taking into consideration the data from earlier publications, we believe that the frequent use of an exoscope might prevent the development of neck and back pain for surgeons in long-lasting procedures due to a unnatural bending posture^2–4,15^. The combination of augmented reality for complex tumor or degenerative cases might be of further interest and was not explored during this study. Additionally, the application of different filters might also be helpful in tumor cases and should be further explored.

## Conclusion

The 3D Aeos exoscope facilitates high quality visualization and three-dimensional depth perception in minimally invasive spinal procedures.

## Data Availability

The data presented in this study are available on request from the corresponding author.
